# The Bristol Royal Infirmary

**Published:** 1907-11-30

**Authors:** 


### THE BRISTOL ROYAL INFIRMARY.

### A SETTLEMENT PRACTICALLY CONCLUDED.

Pending the final settlement of differences between the Medical Staff and the Governors we are glad to learn that there is every reason to believe that a way has been found whereby the difficulties caused by the dissatisfaction of the Honorary Medical Staff with the resolutions adopted by the Governors at the half-yearly meeting in September have been overcome. It may be well, in order to bring out the points clearly, to reprint these regulations, which were carried after an animated discussion by forty-seven votes to twenty-six. They were as follows:—

Resolved : "That all appointments to the honorary staff to be made subsequent to the passing of this rule shall be held subject to the following regulations, in lieu of those contained in Rule 35."

"Rule 36 : No member of the honorary staff shall hold any union or club appointment. No member of the full staff shall hold any other professional public appointment other than professorship or lectureship at any University, college, or school. No member of the assistant staff shall hold any other general hospital appointment, nor more than one special hospital appointment.

"That the full physicians shall limit their practice to medical work. That the full surgeons shall limit their practice to surgical work. That each of the specialists shall limit his practice to his speciality."

During the interval which has elapsed there has been a great deal of discussion and some exhibition of feeling in professional and hospital circles in and around Bristol. Sir George White, the President, has had the grave responsibility of representing the Governors, and he is entitled to credit for having so conducted the business as to bring about an agreement, which is well calculated to conserve the best interests of the Royal Infirmary, Bristol, of its honorary medical staff, and of the medical profession generally.

It is essential to make this quite clear owing to the publication last week of certain statements, before any settlement had been arrived at, which, from their indiscretion, were calculated, however well-intentioned they may have been, to keep the sore open and to render a settlement immensely difficult, to say the least. Anyone who has an intimate knowledge of the services rendered to the Royal Bristol Infirmary by Sir George White and the Governors who have supported him, and especially by the more intelligent members of the medical faculty, must recognise the debt of gratitude that is due to Sir George and his friends, and must realise that, there is probably no one amongst them, who has the interests of the Infirmary and everyone connected with its welfare more at heart, than Sir George White. It was therefore not only indiscreet but wanton to write a hasty, ill-considered, intemperate account of the various stages of the controversy at the very height of the crisis, when all the wiser heads were doing their best to promote peace, and secure a satisfactory and permanent settlement.

Had it not been for the publication of the article referred to we should have been inclined to let the matter stand over without comment until after the special meeting of the Board on December 5 next. As it is we have decided to publish the following alterations to the rules, which have now, we understand, received the approval of the Staff, as well as of Sir George White and his Committee.

The new rule runs as follows:—

"That all appointments to the Honorary Staff to be made subsequent to the passing of this Rule shall [illegible]e held subject to the following regulations, in lieu [illegible] those contained in Rule 35: No member of the [illegible]onorary Staff shall hold any Union or Club appoint[illegible]nt. No member of the Full Staff or of the Assis[illegible]t Staff shall hold any other General Hospital [illegible]ointment, but this rule shall not preclude [illegible]nbers of the Honorary Staff accepting purely consulting and not active appointments at another Hospital. That the Full Physicians shall limit their practice to surgical work. That each of the Specialists shall limit his practice to his specialty."

It will be seen that the result of the negotiations is to eliminate from the original resolution the words: "No member of the full staff shall hold any other professional public appointment other than the professorship or lectureship at any University, College, or School," and to substitute for them the following: "No member of the full staff or of the assistant staff shall hold any other general hospital appointment, or more than one special hospital appointment, but this rule shall not preclude members of the honorary staff accepting purely consulting and not active appointments at another hospital." The effect of the change will be to permit members of the honorary staff, as well as of the assistant staff, to hold one special hospital appointment, whereas it was previously enacted that only members of the assistant staff should be entitled to hold one special hospital appointment. All the members of the staff will in future be precluded from holding any other general hospital appointment, but they will be free to accept purely consulting, as opposed to active appointments at another hospital.

We welcome the amended regulation, for it removes all ambiguity, and clearly states what the Governors intend and what the honorary staff have agreed to.

